# Remarks on the Use of Tobacco

**Published:** 1861-12

**Authors:** D. J. Lyster

**Affiliations:** Brooklyn, N. Y.


Remarks on the Use of Tobacco—*By D. J. Lyster, M. D., of Brooklyn, N. Y.*—It is truly surprising that a single individual can be found, who, after experiencing the distressing sensations almost invariably produced by the first *use of tobacco*, would be willing to risk their recurrence a second time; still more so, that any one should again and again resort to the "noxious weed" until, its immediate effects being lessened by habit, it becomes an article of luxury, from the use of which it is found difficult to refrain. The extreme nausea—pain of the head and vertigo—the cold death-like sweat, and general exhaustion, experienced by the novice in chewing, snuffing, and smoking, we should imagine would be fully sufficient to prevent the use of tobacco from becoming a habit. Yet such is "the folly and infatuation of the human mind," and the power of custom and example, in opposition to prudence and the dictates of nature, that one of the most disgusting productions of the vegetable kingdom, "in all places where it has come," to use the quaint expression of Sir Hans Sloane, "has much bewitched the inhabitants, from the polite European to the barbarous Hottentot."

Did this modern herb possess a tithe of the virtues ascribed to it by Dr. Thorus in his Patologia; did, in fact, the least benefit result to the system from its habitual use, there would then be some reason why, with all its loathsomeness of smell and taste, it should have become so general a favorite. But we know, on the contrary, that all who habituate themselves to its use, sooner or later experience its noxious powers. Tobacco is, in fact, an absolute poison. A very moderate quantity introduced into the system—even applying the moistened leaves over the stomach—has been known very suddenly to extinguish life. The Indians of our country were well aware of its poisonous effects, and were accustomed, it is said, on certain occasions, to dip the points of their arrows in an oil obtained from the leaves, which being inserted into the flesh, occasioned sickness and fainting, or even convulsions and death.

It must be evident to every one, that the constant use of an article possessing such deleterious properties, can not fail at length to influence the health of the person. In whatever form it may be employed, a portion of the active principles of the tobacco mixed with the saliva, invariably finds its way into the stomach, and disturbs or impairs the functions of that organ. Hence, most, if not all, of those who are accustomed to the use of tobacco labor under dyspeptic symptoms. They experience, at intervals, a want of appetite, nausea, inordinate thirst, vertigo, pains and distension of the stomach, disagreeable sensation of the head, tremors of the limbs, disturbed sleep, and are more or less emaciated.

According to Boerhaave, "When this celebrated plant was first brought into use in Europe, it was cried up for a certain antidote for hunger, but it was soon observed that the number of hypochondriacal and consumptive people were greatly increased by its use." Dr. Cullen informs us that he has observed several instances, in which the excessive use of tobacco in the form of snuff, has produced effects similar to those occurring in persons from the long continued use of wine and opium, that is, "loss of memory, fatuity, and other symptoms of a weakened or senile state of the nervous system induced before the usual period." The almost constant thirst occasioned by smoking and chewing has, in numerous instances, it is to be feared, led to the intemperate use of ardent spirits. This thirst can not be allayed by water; for no insipid liquor will be relished after the mouth and throat have been exposed to the stimulus of the smoke or juice of the tobacco; a desire, of course, is excited for strong drinks, which soon leads to intemperance and drunkenness. The use of snuff destroys entirely the sense of smell, and injures materially the tone of the voice; while chewing and smoking vitiate the sense of taste. Hence, those who make use of tobacco to any extent, have certainly one, and frequently two of the external senses less perfect than other individuals. But this is not all; Polypus of the nose, and *other* serious affections, have been traced to the use of snuff.

Sir John Pringle, who, we are informed, was very liberal in its use, experienced in the evening of his days a tremor of his hands, and a defect of memory. Being in company with Dr. Franklin, at Paris, he was requested by the Doctor to observe that the former complaint was very common to those people of fashion who were great snuffers. Sir John was led by this remark to suspect that his tremors were occasioned by his excessive use of snuff. He therefore immediately left it off, and soon afterwards the tremor of his hands disappeared, and at the same time he recovered the perfect exercise of his retentive faculties.

Cases could be mentioned in which epilepsy, consumption, and other serious diseases have been brought on in young people by the excessive use of tobacco. I have known myself individuals in whom very severe and dangerous affections of the stomach, tremors of the limbs, and great emaciation were referrible to excessive smoking and chewing, and which were removed only after these habits were entirely relinquished.

One or two of these cases, I am sorry to say, occurred in females, from the filthy practice of chewing snuff; and in a class of society where it was to be hoped a refinement of taste and exalted notions of female delicacy, would forever have precluded the introduction of so detestable and pernicious a habit.—*American Medical Times.*

